# Markers of stem cells in human ovarian granulosa cells: is there a clinical significance in ART?

**DOI:** 10.1186/1757-2215-5-36

**Published:** 2012-11-20

**Authors:** Michail Varras, Theodora Griva, Vasileios Kalles, Christodoulos Akrivis, Nikolaos Paparisteidis

**Affiliations:** 1 Third Department of Obstetrics and Gynecology, “Elena Venizelou” General Maternity Hospital, Platonos 33, Politia (Kifisia), Athens, 14563, Greece; 2 Medical School, University of Athens, Athens, Greece; 3 Naval and Veterans Hospital of Athens, Athens, Greece; 4 Department of Obstetrics and Gynecology, “G. Chatzikosta” General State Hospital, Ioannina, Greece; 5 IVF Unit, “Elena Venizelou” General Maternity Hospital of Athens, Athens, Greece

**Keywords:** Granulosa cells, Infertile patients, Oct-4, DAZL, Stem cells, IVF

## Abstract

**Background:**

The purpose of the study was to determine the incidence of gene expression of Oct-4 and DAZL, which are typical markers for stem cells, in human granulosa cells during ovarian stimulation in women with normal FSH levels undergoing IVF or ICSI and to discover any clinical significance of such expression in ART.

**Methods:**

Twenty one women underwent ovulation induction for IVF or ICSI and ET with standard GnRH analogue-recombinant FSH protocol. Infertility causes were male and tubal factor. Cumulus–mature oocyte complexes were denuded separately and granulosa cells were analyzed for each patient separately using quantitative reverse-transcription–polymerase chain reaction analysis for Oct-4 and DAZL gene expression with G6PD gene as internal standard.

**Results:**

G6PD and Oct-4 mRNA was detected in the granulosa cells in 47.6% (10/21). The median of Oct-4 mRNA/G6PD mRNA was 1.75 with intra-quarteral range from 0.10 to 98.21. The OCT-4 mRNA expression was statistically significantly correlated with the number of oocytes retrieved; when the Oct-4 mRNA expression was higher, then more than six oocytes were retrieved (p=0.037, Wilcoxon rank-sum). No detection of DAZL mRNA was found in granulosa cells. There was no additional statistically significant correlation between the levels of Oct-4 expression and FSH basal levels or estradiol peak levels or dosage of FSH for ovulation induction. No association was found between the presence or absence of Oct-4 mRNA expression in granulosa cells and ovarian response to gonadotropin stimulation. Also, no influence on pregnancy was observed between the presence or absence of Oct-4 mRNA expression in granulosa cells or to its expression levels accordingly.

**Conclusions:**

Expression of OCT-4 mRNA, which is a typical stem cell marker and absence of expression of DAZL mRNA, which is a typical germ cell marker, suggest that a subpopulation of luteinized granulosa cells in healthy ovarian follicles (47.6%) consists of stem cells, which are not originated from primordial germ cells. Absence of Oct-4 gene expression in more than half of the cases means probably the end of the productive journey of these cells, towards the oocyte.

## Background

The adult human ovary is composed of various cell types. Within the follicle, paracrine communications between oocyte, granulosa cells and thecal cells are critical for normal follicular development and further fertilizability [1-6]. The paracrine factors, maintained by the gonadotrophins, allow for a dialogue between oocyte and granulosa cells through gap junctions, promote granulosa cells differentiation, affect the pattern of gene expression and contribute to oocyte maturation [5,7-13]. Kossowska-Tomaszczuk et al. (2009) demonstrated the presence of multipotent granulosa cells, which survive in the presence of leukemia-inhibiting factor (LIF) and suggested that the follicular granulosa cells consist of subpopulations of differentiated and less differentiated cells [14]. Also, some studies have showed regeneration of oocytes from putative germ cells in bone marrow and peripheral blood or even from somatic stem cells [15,16]. It is not clear yet if the follicular granulosa cells could serve as a new source for germ cells.

At present, there are no morphological or physiological features of oocytes that can predict whether IVF fertilization will be successful, or whether there is a need for ICSI [17]. Moreover, in the field of assisted reproduction, the selection of embryos with high implantation potential remains one of the major goals in order to transfer one embryo and avoid therefore the adverse outcomes related to multiple pregnancies [5]. However, the study of human oocytes is limited by their complex availability and their low number, while granulosa are easily available since they are always discarded before ICSI procedures [5]. Recently the use of ICSI in cases unrelated to male factor infertility has increased greatly at ART facilities [17-19]. Such general use of ICSI, however, raises concerns because of its greater costs, such as the increase in the risk of transmitting chromosomal anomalies or imprinting disorders [20-22], although it is not clear whether these risks are due to the procedure or to the factors causing male infertility [17,23]. In view of the concept of the beneficial effect of granulosa cells on oocyte maturation, the need for greater understanding of the mechanisms involved in late oogenesis and the need for independent prognostic markers of better outcomes using conventional IVF for couples with non-male factor infertility, we focused on Oct-4 and DAZL as target genes in luteinized granulosa cells of the human ovaries.

Octamer-binding transcription factor-4 (Oct-4, also known as POU5F1) is a member of Pit-Oct-Unc (POU) transcription factor family [24,25]. Oct-4 is expressed in whole embryos at different stages of development, embryonic stem (ES) cells, germ cells, embryonal and germ-cell tumors, such as embryonal carcinoma (EC), testicular carcinoma *in situ*, dysgerminoma, seminoma, and embryonal carcinoma component of nonseminomatous cell tumor [26-35]. Embryoid bodies from mouse stem cells are found to express Oct-4 gene [36]. Also, during early mouse preimplantation development, Oct-4 is expressed in all blastomeres and at blastocyst stage it is confined to inner cell mass and is down-regulated in trophectoderm. After implantation, Oct-4 expression is restricted to the epiblast, is down-regulated during gastrulation, and is later confined to primordial germ cells [29,33,37,38]. During fetal, newborn, and adult spermatogenesis, Oct-4 expression is limited to most undifferentiated spermatogonia [38]. In fetal ovaries, Oct-4 expression ceases soon after the germ cells have entered meiosis, it reappears before the beginning of oocyte growth and then is maintained throughout folliculogenesis [33]. Oct-4 in co-operation with other factors (e.g. FOXD3, SOX2, STAT3) regulates tissue- and cell-specific transcription via the consensus motif ATGCAAAT [30,39-42]. The Oct-4 gene contains the proximal enhancer (PE) and the distal enhancer (DE) that are important for Oct-4 cell type-specific expression, and the four conserved domains CR1-CR4, that are important for Oct-4 basal expression [43]. Oct-4 expression is restricted to pluripotent cells, while the loss of Oct-4 expression may be associated with loss of pluripotentiality [41]. Oct-4 is involved in the self-renewal of undifferentiated embryonic stem cells and is therefore used as a marker of embryonic stem cells [25,28,39,40,44].

The protein DAZL (DAZ-like) is RNA binding protein which is a member of the DAZL (deleted in azoospermia like) family which also includes BOULE and DAZ. The genes of the DAZL family encode proteins with a highly conserved RNA-binding motif (RNA recognition motif, RRM) and a unique DAZ repeat of 24 amino acids. These proteins are believed to function in the post-transcriptional regulation of messenger RNA (mRNA) expression [45-48]. The proteins of the DAZL family are located to nucleus and cytoplasm of the fetal germ cells [49]. In the male, DAZL is expressed during spermatogenesis in gonocytes, spermatogonia and primary spermatocytes. During meiosis, DAZL is translocated from the nucleus of the spermatogonia into the cytoplasm of secondary spermatocytes, spermatids and spermatozoa [50-53]. During oogenesis, human DAZL is expressed in the cytoplasm of oogonia and in developing follicular oocytes in fetal and adult ovaries [54-58]. Targeted deletion of the *DAZL* gene results in germ cell loss in both males and females in humans and mice [52]. In *DAZL* −/− females germ cell loss occurred in fetal ovaries at the time of meiotic entry and adult ovaries did not contain oocytes [52,59]. In *DAZL* −/− males the pattern of germ cell loss is variable and in some studies it has been reported to occur during fetal life, whilst in others it was associated with spermatogonial differentiation or entry to meiosis [46,48,51,60,61]. In human males, decreased DAZL expression has been reported in testes which produce little or no sperm [62] and may be associated with primary amenorrhea or premature ovarian failure in women [54,63]. Not unique to germ cells, DAZL transcripts are also found in somatic Sertoli cells of the gonad [63-65]. DAZL has also been reported to be expressed in human and mouse granulosa cells [52,54], human theca interna cells [55] and in the granulosa-luteal cells of human corpus lutea [59,66], but this remains controversial [67]. In addition, DAZL and Oct-4 gene expression has been found in human amniotic fluid cells promising the potential of these cells as a multipotent cell course for regenerative somatic cell therapy, and very recently DAZL expression has been found in mouse bone marrow mesenchymal cells [49,68].

The purpose of this study was to investigate whether human luteinized follicular granulosa cells contain cells expressing the transcription factors Oct-4 or DAZL and whether there is any relationship between the expressed genes and the infertile clinical features and outcomes after IVF or ICSI and embryo transfer (ET).

## Methods

### Participants

21 women who had undergone to IVF or ICSI and ET were enrolled in the study in order to determine the expression of Oct-4 and DAZL mRNA in their ovarian follicular granulosa cells. Among them, 13 cases (62%) underwent IVF due to tubal disease and 8 cases (38%) underwent ICSI due to male infertility. The patients were submitted to the same ovulation protocol. Women with history of diabetes mellitus and/or polycystic ovarian syndrome (PCOS), as well as women with endometriosis were excluded from the study. The study received approval from the hospital’s ethics committee, and written informed consent was obtained from the participants of this study. Details for the hormone assays are given in the recent manuscript by Varras et al. [69].

### Interventions

The protocol for controlled ovarian hyperstimulation and follicle monitoring, have previously been described [69]. In order to determine Oct-4 and DAZL mRNA expression, cumulus cells were collected during oocyte retrieval. The cells were segregated from cumulus-mature oocyte complexes (CMOCs) through the process of stripping using a fire-polished tip glass pipette. The selection of the cumulus-mature oocyte complexes (CMOCs) before cell isolation was random. RNA was extracted using the RNeasy Micro Kit (Qiagen, Valencia, CA, USA). RNA extraction was conducted according to manufacturer’s protocol. The extracted RNA was a product of cumulus cells pooled from several CMOCs and not only from the oocytes that proceeded to embryo transfer. Moreover, RNA concentration of each sample was determined by spectrophotometry and its quality was evaluated by agarose gel electrophoresis. cDNA preparation was performed using 20 ng aliquots of total RNA extracted. RNA was reverse-transcribed using 0.5 mM dNTP mix (Ambion, Austin, Tx, USA), 5 μM oligo dT Primer (Ambion, Austin, Tx, USA), 1xRT buffer (Ambion, Austin, Tx, USA), 80 U ribonuclease inhibitor (Invitrogen Life Technologies), 1600 U M-MLV reverse transcriptase (Invitrogen Life Technologies) and nuclease free water (Ambion, Austin, Tx, USA) to a total volume of 40 μl. The reactions were carried out in Mastercycler (Eppendorf) with the following conditions: 80°C for 3 min, 42°C for 60 min and 92°C for 10 min. The resulting cDNAs were stored at −20°C.

**Figure 1:**
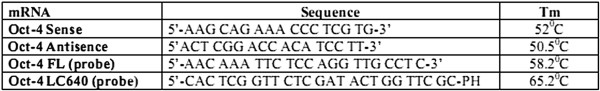
**mRNA sequences for Oct-4 genes.** Sense and antisense mRNA sequences for Oct-4; (Tm: Melting temperature).

**Figure 2:**
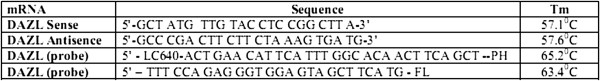
**mRNA sequences for DAZL genes.** Sense and antisense mRNA sequences for DAZL genes; (Tm: Melting temperature).

The expression of Oct-4 and DAZL mRNA in luteinized granulosa cells were assessed by real-time PCR using sense and antisense primer pairs and hybridization probes particularly synthesized by TIB-MOLBIOL for this study. Primers (Sense and Antisence) and fluroscent Probes (FL and LC) sequences are seen in Figures 1 and 2. The specific primers and probes were used at concentrations 20 pmol/μl for each reaction. To determine the steady amount for Oct-4 and DAZL mRNA levels in granulosa cells, a quantitative competitive PCR (QC RT-PCR) was developed using a LightCycler 480 (Roche). Samples were run in duplicate and no template controls were included in all runs to exclude possible DNA contaminations. The 20μl RT-PCR reaction mixture contained 4μL Light Cycler 480 Genotyping Master (Roche), 0.5μL of each primer, 0.2 μl of each probe, 9.6 μl H_2_O of LightCycler 480 Genotyping Master (Roche) and 5μl of cDNA. The expression of Oct-4 and DAZL genes were normalized with the data for G6PD (Light Mix Kint G6PD, TIB MOLBIOL). The respective quantitative ratio was determined by the density of each target to the internal standard: Oct-4mRNA/G6PDmRNA and DAZLmRNA / G6PDmRNA. PCR was performed on Light Cycler 480 (Roche) with the following parameters: one cycle at 95°C for 10-sc for pro-incubation, 40 cycles for amplification (95°C for 10 sc, 56°C for 20 sc, 72°C for 10 sc) and one cycle at 4°C for cooling.

### Statistical analyses

All statistical analyses were performed using the STATA 9 statistical software. Differences between qualitative/categorical variables were evaluated with the Fisher’s exact test. Non-parametric Wilcoxon rank-sum and Kruskal-Wallis tests were used to compare differences of quantitative variables between categories of qualitative variables. The Spearman rank correlation coefficient (Spearman’s rho) was used to analyze the relationship between two different values. Multiple linear regression analysis and multiple logistic regression analysis were performed to determine the relationship between the variables. A *p* value <0.05 was considered statistically significant.

## Results

### Patients characteristics

The average age of the patients was 35.52 ± 4.06 years, the average BMI was 23.08 ± 4.35, the average basal FSH levels (IU/l) were 6.66 ±2.52 and the average prolactin (PRL) levels (ng/ml) were 12.97±7.32. The Table 1 presents additional demographic and clinical parameters.

**Table 1:** Patient characteristics

**Characteristics**	**Median (IQR)**
Infertility duration (years)	4.00 (3.00–6.50)
Serum LH (IU/L)	4.60 (3.36–8.40)
Total gonadotropin dose (IU)	2,875.00 (2,037.50–3,587.50)
Duration of stimulation (days)	10.00 (9.00-11.00)
Serum estradiol on the 5^th^ day of rFSH administration (pg/ml)	500.00 (309.00 – 758.00)
Serum estradiol on the day of hCG administration (pg/ml)	1,910.00 (1,570.00–2,467.00)
Number of follicles aspirated	8.00 (6.00-10.00)
Grade 2 embryos	0.00 (0.00-2.00)
Grade 3 embryos	4.00 (4.00-6.00)
High quality embryo ratio (%)	100.00 (66.67 – 100.00)

Patients’ demographics and clinical parameters.

Fertilization was successful in all cases in the laboratory (21/21). A pregnancy test was positive in 29% (n=6) of cases and a clinical pregnancy was confirmed in 19% (n=4) of women. No multiple pregnancies were observed, while a first trimester abortion and an ectopic pregnancy occurred in 1 case each. Positive pregnancy test was found in 4 cases in the ICSI group and in 2 cases in the IVF group. Clinical pregnancy was found in 2 cases in each group. The median for age in the ICSI group was 37.0 (IQR: 34.5-38.5), while in the IVF group was 36.0 (IQR: 35.0-36.0). The median for PRL in the ICSI group was 11.1 (IQR: 7.1-13.8) and in the IVF group was 12.1 (IQR: 8.8-21.9). The median for estradiol levels (pg/ml) the fifth day of rFSH administration in the ICSI group was 449.5 (IQR: 309.0-638.0), while in the IVF group was 508.0 (IQR: 350.0-933.0). The median number of oocytes retrieved in the ICSI group was 8.5 (IQR: 6.0-9.5), while in the IVF group was 7.0 (IQR: 6.0-9.0).

### Incidence of Oct-4 gene expression and absence of DAZL gene expression in granulosa cells

Oct-4 gene expression in luteinized granulosa cells in women that underwent IVF or ICSI was observed in 48% of the studied cases (10 out of 21). In the 10 cases, in which the Oct-4 gene was expressed, the median Oct-4 mRNA/G6PD mRNA was 1.75 (quartile range 0.10 – 98.21). On the other hand, no DAZL gene expression was observed in any of the 21 cases studied.

### Expression of Oct-4 gene in granulosa cells according to clinical parameters

There were no significant differences among the presence or absence of Oct-4 gene expression and age, BMI, years and causes of infertility, previous assisted reproduction attempts, basal serum FSH and LH levels, serum levels of PRL, serum oestradiol levels on the fifth day of rFSH administration and on the day of hCG administration, the total dose of rFSH, the duration of treatment, the type of assisted reproduction, the number of follicles aspirated, the total number of oocytes retrieved, the number of mature oocytes retrieved, the mature oocytes ratio (<60%, 60-75% and ≥75%), the embryo grade, the positive pregnancy test and the existence of clinical pregnancy (Tables 2 and 3).

**Table 2:** Oct-4 expression – baseline characteristics and outcomes

	**Οct-4 expression**		p-value*
	**No n (%)**	**Yes n (%)**	
Infertility Cause			0.864
*Male*	4 (36.4)	4 (40.0)	
*Fallopian tube*	7 (63.6)	6 (60.0)	
Previous IVF/ICSI cycles			0.525
*No*	9 (81.8)	7 (70.0)	
*Yes*	2 (18.2)	3 (30.0)	
Embryos grade			0.296
*3*	9 (81.8)	5 (50.0)	
*3 + 2*	1 (9.1)	3 (30.0)	
*2*	1 (9.1)	2 (20.0)	
Positive pregnancy test			0.269
*No*	9 (81.8)	6 (60.0)	
*Yes*	2 (18.2)	4 (40.0)	
Clinical pregnancy			0.916
*No*	9 (81.8)	8 (80.0)	
*Yes*	2 (18.2)	2 (20.0)	

*Statistical Test.

Presence or absence of Oct-4 mRNA expression in correlation with the patients’ baseline characteristics and the outcomes from the assisted reproduction technologies.

**Table 3:** Oct-4 expression – cycle characteristics

	**Οct-4 expression**		p-value*
	**Yes median (IQR)**	**No median (IQR)**	
Age (ys)	36.00 (35.00, 38.00)	36.00 (30.00, 37.00)	0.520
BMI	23.20 (19.80, 24.60)	22.80 (20.10, 26.30)	0.778
Serum FSH (IU/L)	6.30 (5.80, 7.50)	5.90 (4.30, 8.10)	0.545
SerumLH (IU/L)	4.50 (3.40, 4.60)	5.70 (3.40, 9.70)	0.165
SerumPRL (ng/ml)	11.40 (9.70, 15.30)	10.40 (5.50, 25.10)	0.790
Total gonadotropin dose	2,675.00 (1800.0, 3200.0)	3,112.50 (2325.00, 3675.00)	0.364
Duration of stimulation (ds)	10.00 (9.00, 11.00)	10.00 (9.00, 11.00)	1.000
Serum estradiol on the 5^th^ day of rFSH administration (pg/ml)	500.00 (350.00, 758.00)	455.00 (180.00, 890.00)	0.805
Serum estradiol on the day of hCG administration (pg/ml)	1,800.00 (1,200.00, 2,025.00)	2,283.50 (1,828.00, 2,617.00)	0.149
No of follicles aspirated	8.00 (6.00, 10.00)	8.50 (6.00, 10.00)	0.972
No of oocyte retrieved	8.00 (6.00, 9.00)	8.00 (6.00, 10.00)	0.887
Mature oocytes ratio (%)	75.00 (67.00, 80.0)	68.5 (67.0, 78.0)	0.415

*Statistical Test.

Presence or absence of Oct-4 mRNA expression in correlation with the patients’ cycle characteristics.

**Figure 3:**
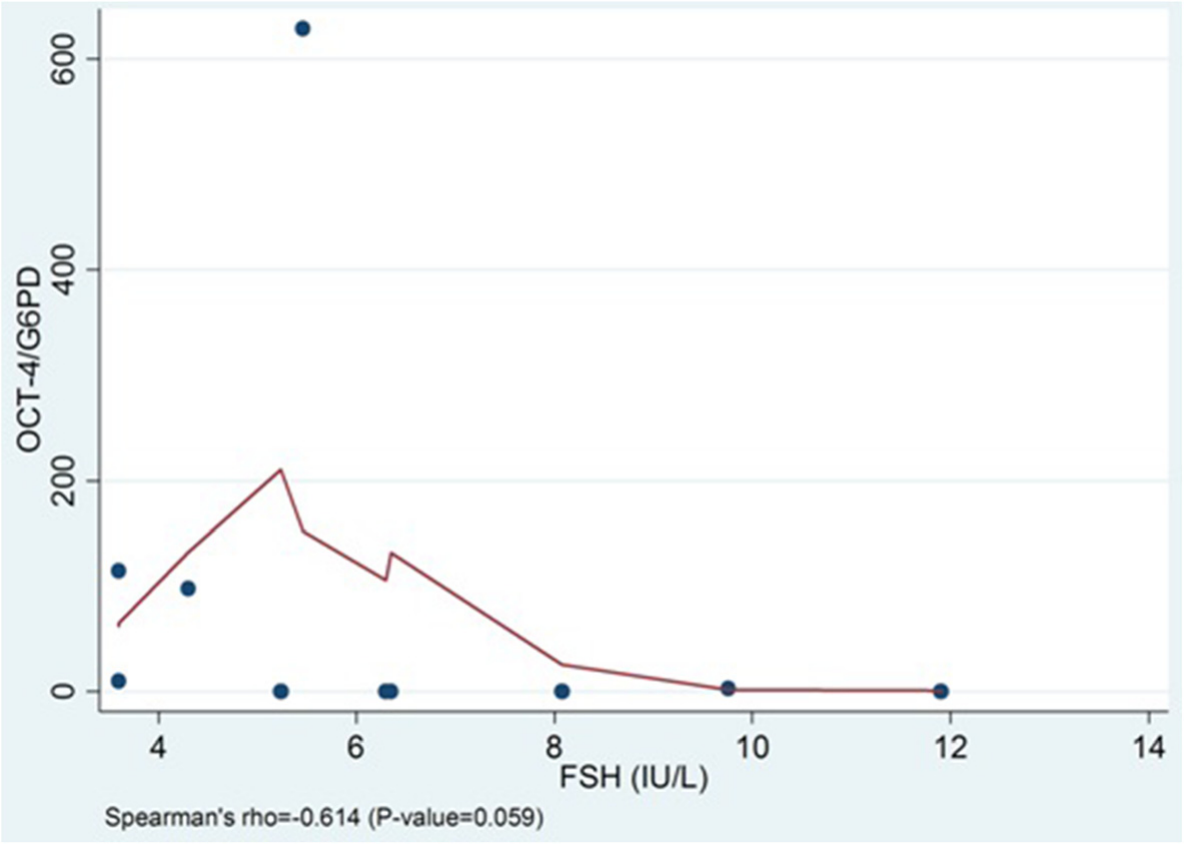
**Levels of Oct-4 expression and FSH levels.** There is a trend for negative correlation between Oct-4 mRNA expression levels and the FSH basal levels, however it did not reach statistical significance (Spearman’s rho=−0.614, p= 0.059).

**Figure 4:**
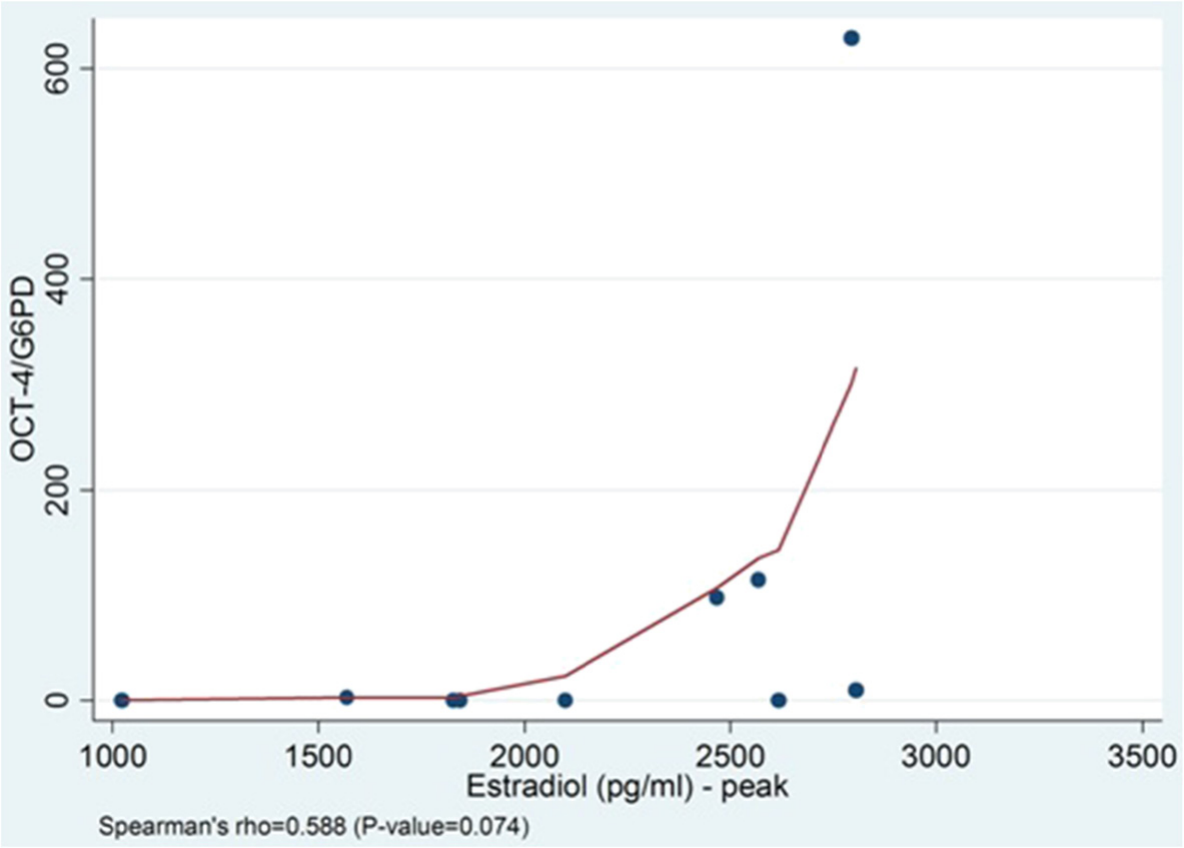
**Levels of Oct-4 expression and peak estradiol levels.** There is a statistical non-significant trend for positive correlation between Oct-4 mRNA expression levels and the estradiol peak levels after hCG administration (Spearman’s rho=0.588, p=0.074).

In addition, the above parameters were examined in relation to the levels of Oct-4 mRNA expression in granulosa cells. There was a trend for negative correlation between the basal serum levels of FSH and the Oct-4 mRNA expression levels, however it did not reach statistically significantly (Spearman’s rho=−0.614, p=0.059) (Figure 3). Also, a non-significant trend for positive correlation was recorded between the peak levels of estradiol on the day of hCG administration and the levels of Oct-4 mRNA expression (Spearman’s rho=0.588, p=0.074) (Figure 4). The Oct-4 mRNA expression was statistically significantly correlated with the number of oocytes retrieved; when the Oct-4 mRNA expression was higher, then more than six oocytes were retrieved (p=0.037, Wilcoxon rank-sum). No other significant correlation between Oct-4 mRNA expression and total gonadotropin dose and number of mature oocytes aspirated was found.

Multiple regression analysis was performed to examine possible contributing factors. When embryo grade (grade 3, grade 3+2, grade 2) was used as the dependent variable, whereas age (ys), PRL (ng/ml), estradiol levels the fifth day of rFSH administration (pg/ml) and levels of Oct-4/G6PD mRNA expression as independent variables, only estradiol levels on the fifth day of rFSH administration and total rFSH dose administrated were significantly related to the embryo grade (p=0.040 and p=0.029 respectively) (Table 4). Additionally, when the clinical pregnancy was used as dependent variable and the above parameters as independent variables no correlations were found to be statistically significant (Table 5).

**Table 4:** Embryo grade – cycle characteristics

**Characteristics**	**Embryo grade**			*p *value*
	**Grade 3**	**Grade 3+2**	**Grade 2**	
Age (years)	36.00 (35.00, 37.00)	37.00 (33.00, 39.50)	33.00 (27.00, 37.00)	0.462
FSH (IU/L)	6.00 (4.90, 6.80)	7.60 (4.50, 11.40)	6.30 (6.10, 8.10)	0.607
PRL (ng/mL)	11.40 (9.30, 15.90)	6.00 (3.60, 21.90)	13.80 (4.90, 16.10)	0.581
Total gonadotropin dosage	2,700.00 (2,275.00, 3,150.00)	2,387.00 (1,525.00, 3,300.00)	4,000.00 (3,675.00, 4,050.00)	0.029
Serum estradiol, 5^th^ day of rFSH administration (pg/mL)	546.50 (450.00, 933.00)	455.00 (250.50, 633.50)	157.00 (63.00, 309.00)	0.040
Serum estradiol peak (pg/mL)	1,967.50 (1,700.00, 2,467.00)	2,182.50 (1,385.00, 2,800.00)	1,828.00 (1,023.00, 2,022.00)	0.556
Number of oocytes retrieved	8.00 (6.00, 9.00)	8.50 (6.00, 10.50)	8.00 (4.00, 9.00)	0.713
Mature oocyte ratio (%)	71.00 (67.00, 78.00)	75.00 (63.50, 85.50)	75.00 (50.00, 78.00)	0.887
Oct-4/G6PD	0.10 (0.10, 98.20)	10.20 (3.10, 628.60)	0.20 (0.00, 0.40)	0.280

*Statistical Test.

Embryo grade in correlation with patients’ cycle characteristics and levels of Oct-4/G6PD genes expression.

**Table 5:** Clinical pregnancy – cycle characteristics

**Characteristics**	**Clinical Pregnancy**		*p *value*
	**No Median [IQR]**	**Yes Median [IQR]**	
Age (years)	36.00 (33.00, 37.00)	37.50 (35.50, 41.50)	0.238
FSH (IU/L)	6.30 (5.60, 8.00)	4.20 (3.80, 5.50)	0.065
PRL (ng/mL)	10.00 (7.10, 15.30)	16.80 (11.40, 25.10)	0.137
Total gonadotropin dosage	2,987.50 (2,037.50, 3,662.50)	2,512.00 (1,725.00, 3,112.50)	0.345
Serum estradiol, 5^th^ day of rFSH administration (pg/mL)	500.00 (309.00, 758.00)	503.50 (403.50, 729.50)	0.754
Serum estradiol, peak (pg/mL)	1,845.00 (1,570.00, 2,317.00)	2,188.50 (1,466.50, 2,636.00)	0.687
Number of oocytes retrieved	7.00 (6.00, 90.00)	9.00 (7.50, 9.50)	0.468
Mature oocyte ratio (%)	75.00 (67.00, 78.00)	68.50 (67.00, 76.50)	0.719
Oct-4/G6PD	0.30 (0.10, 58.80)	54.20 (10.20, 98.20)	0.296

*Statistical Test.

Clinical pregnancy in correlation with patients’ cycle characteristics and levels of Oct-4/G6PD genes expression.

## Discussion

The present study showed the expression of Oct-4 in luteinized granulosa cells of women that underwent IVF or ICSI. To the best of our knowledge, this is the first report which determined the frequency of Oct-4 in these cells and investigated the correlations between the presence or absence and the levels of Oct-4 gene expression with infertility clinical background and the assisted reproduction outcomes [14]. Kossowska et al. [14], in order to get past the fact that the status of infertility of individual patients might affect the function of granulosa cells, pooled granulosa cells from all the different patients who studied and examined the expression of POU5F1 in the sum of cells. Therefore, the present study gives an additional estimation on the frequency of expression of the Oct-4 in luteinized granulosa cells, which is detected in almost half of the cases. The expression of Oct-4, in granulosa cells reported herein, is in agreement with the previous findings from Kossowska et al. [14], and reinforces the implication that the granulosa cells of healthy follicles are not uniform but consist of two distinct subpopulations of differentiated and less differentiated cells, with the less differentiated cells being capable of mitosis. The expression of Oct-4 supports the perception that this subpopulation has significant characteristics such as pluripotent and self-renewal capabilities. Kossowska et al. (2009) in order to confirm the multipotency of granulosa cells examined typical mesenchymal stem markers and found that CD29, CD44, CD90, CD105, CD117, and CD166, but not CD73, were expressed by substantial subpopulations of granulosa cells. Prolonged culture of luteinizing granulosa cells in medium supplemented with LIF allowed the selection of less differentiated granulosa cells, which exhibited a certain degree of plasticity, as they could be differentiated *in vitro* into three distinct lineages: neuronal, chondrocytic, and osteoblastic, all normally not found in healthy ovarian follicles. Moreover, follicle-derived stem cells were able to survive when transplanted into the backs of immunoincompetent mice, *in vivo* generating tissues of mesenchymal origin [14].

Recently, the ovarian surface epithelium of adult human females was reported to be a source of germ cells. Bukovsky et al. [70] reported the presence of bipotent progenitor cells and Virant-Klun at al [71] reported stem cells in the adult human ovaries that develop into oocyte-like and parthenote-like structures [70,71]. Also, Parte et al. [72] found pluripotent gene transcripts of Oct-4, Nanog, Sox-2, TERT and STAT-3 in ovarian surface epithelium, while germ cell markers like c-Kit, DAZL, GDF-9, VASA and ZP4 were localized in oocyte-like structures. In addition, the bone marrow mesenchymal stem cells were reported to be a source of germ cells [70,73]. Another important query raised was why the germline stem cell precursors fail to maintain ovarian function with advancing age [74]. It is possible that age associated changes in the immune system could be responsible for the termination of neo-oogenesis and follicular renewal *in vivo*[75]. The DAZL gene expression is a marker for pluripotent stem cells characteristics in germ cells. However, the expression of DAZL gene in human granulosa cells remains controversial [68]. Hence, we examined the DAZL gene expression in the luteinized granulosa cells of women undergoing ovulation stimulation for assisted reproduction, in order to clarify this controversial field. However, in no case was the DAZL gene observed to be expressed. This important result is in concordance with previous findings reported by Kossowska et al. [14], who did not observe markers for pluripotent stem cells characteristic of the germ cells, such as nanog, vasa and stella. Therefore, the hypothesis that the stem cells found in granulosa cells cannot be differentiated in germ cells is strongly supported because of the lack of DAZL, nanog, vasa and stella gene expression. In addition, Stefanidis et al. (2009) found that at least in murine induced embryoid bodies there is simultaneous expression of oxytocin receptors and germ cell markers (DAZL) in many cells (expressing Oct-4). Thus the authors concluded that, the oxytocin might indeed be a molecule playing a leading role in germ cell determination [36].

A more accurate embryo selection in human assisted reproduction is needed to optimize and reduce the number of embryos to be transferred into the uterus in order to achieve the best balance between reducing the risk of multiple gestations and maximizing the probability of pregnancy. Current selection methods are based mainly on morphological and developmental criteria and include the speed of cell division (number of blastomeres at any given stage of development), the regularity of cell division and the degree of fragmentation [76,77]. Moreover, in some countries, not all retrieved oocytes can be fertilized due to legal limitations; for example the Italian legislation allows only three oocytes for each patient to be fertilized [78]. In such situations, predicting embryo quality is even more challenging because the time when the predictive evaluation can be performed is limited to the interval between oocyte retrieval and fertilization. This has prompted the search for additional parameters that can support morphological and metabolic evaluations of the oocyte in order to appropriately select those that have the greater chance of fertilization and development. In this respect, the analysis of granulosa cells is a good approach for providing such supplementary information. During follicular development, the granulosa cells differentiate into two distinct phenotypes, the mural population lining the follicular antrum and the cumulus population enclosing the oocyte. The former is essential for oestrogen production and follicular rupture, while the latter is closely associated with oocyte development. Cumulus cell function is in part regulated by oocyte derived factors and, in turn, contributes to oocyte maturation and subsequent developmental potential [9,77]. The cumulus cells, in fact, are closely connected to the oocyte through a gap junction network during follicular development and ovulation [79,80]. Following the preovulatory LH surge, the intercellular connections are broken, but cumulus cells undergo a process of expansion that continues to bind these cells to the oocyte throughout the ovulatory process and in subsequent fertilization. Characteristics of the expansion include the secretion of a hyaluronic acid rich matrix by the granulosa cells and expression of a number of other proteins required for matrix formation and retention [76,80-84]. In our study we investigated the expression of Oct-4 in follicular granulosa cells of women undergoing assisted reproductive technologies and we think this was a very intriguing approach. The participating patients in the IVF group gave written consent for some of the cumulus–mature oocyte complexes (CMOCs) to be used only for the study. Therefore the cumulus–mature oocyte complexes (CMOCs) were randomly selected and manually denuded separately using a fire-polished tip glass pipette. These granulosa cells were analyzed for each patient separately, but the corresponding mature oocytes were not fertilized because for the IVF procedure the presence of cumulus cells is needed. Only some cumulus – mature oocytes complexes were donated and the cumulus cells were taken only from the mature oocytes per patient. In case of ICSI method, the cumulus-mature oocytes complexes (CMOCs) were manually denuded from granulosa cells using a fire-polished tip glass pipette. Granulosa cells from all the mature oocytes per patient were collected together. ICSI was performed only in oocytes that were morphologically confirmed to be in metaphase II with the first polar body extruded (mature oocytes). In case of the ICSI the cumulus cells were taken only when they surrounded the mature oocytes and these were the ones we analyzed. Therefore, the populations of both groups were homogenous as the cumulus cells in the ICSI group were only from mature oocytes and not from mature, immature and degenerated oocytes. In this way, we examined the expression of Oct-4 mRNA in granulosa cells from each patient separately in correlation with duration of ovulation induction, number of follicles aspirated, number of oocytes retrieved, number of mature oocytes retrieved, embryo grade and clinical pregnancy and found a clear clinical significance only for the number of oocytes retrieved suggesting that Oct-4 expression positively affects the oocyte development during ART. The possibility for stem cell contamination during egg retrieval or granulosa cells collection and possible Oct-4 expression should be excluded because of the absence of DAZL gene expression, which is typically expressed in gametes. Our population included only patients with male or tubal factor infertility. It would be interesting if further studies investigated expression with any clinical significance of Oct-4 gene in granulosa cells of patients with diminished ovarian reserve (DOR) as such population was not included in our study. Also, it would be interesting if more studies validated the expression of Oct-4 using Western blot analysis and immune-fluorescence on granulosa cells in order to overcome possible limitations. Western blot analysis and immune-fluorescence on granulosa cells are going to reinforce our findings. Studying any clinical significance in ART of the expression of stem cell markers in luteinized granulosa cells is a new field of knowledge and according to our knowledge the present study is the first one. Previous studies on the same field were not done before. More of the studies were performed to correlate the impact of apoptosis or survival factors in granulosa cells with ART parameters and outcome. Nakahara et al. (1997) suggested that when the quality of eggs is small, measured by apoptosis in granulosa cells, then the eggs are more likely to be fertilized by ICSI compared to IVF method [85]. However, Clavero et al. (2003) found that the rate of apoptosis in granulosa cells was not associated with the maturity of the oocyte and the ability for fertilization in ICSI or the quality of follicles during ovulation induction [86]. Greenseid et al. (2011) found that IGF1, IGF2 and their receptors are down regulated in ovarian granulosa cells of women with diminished ovarian reserve (DOR) compared to those with normal ovarian reserve (NOR) undergoing in vitro fertilization (IVF) [87]. Also, Fujino et al. (2008) studied the expression of survivin gene in granulosa cells from infertile Japanese patients and found that the gene expression levels of survivin in patients with endometriosis were significantly lower than in patients with male factor infertility. The gene expression levels of survivin in total pregnant patients were higher than those in total non pregnant patients [88]. Moreover, Varras et al. (2012) studied only normal women (male factor infertility) and women with tubal factor infertility who underwent IVI or ICSI and embryo transfer [69]. Women with endometriosis or polycystic ovarian syndrome were not included in their study since endometriosis and androgens promote apoptosis [85,89]. Varras et al. found a statistically significant increased expression of survivin in granulosa cells of women who had tubal factor infertility compared to normal women (male factor infertility). Therefore, it seems that survivin acts a protective role in the ovarian micro-environment. It is possible that survivin might try to protect ovaries, with possible influenced perfusion due to ipsilateral salpingectomy. In cases with tubal inflammation or hydrosalpinges survivin might try to protect the ovaries from follicular apoptosis in a paracrine environment [69]. The authors suggested their hypothesis about the levels of survivin mRNA expression in ovarian granulosa cells in tubal factor infertility. Some patients in the subpopulation of women with tubal factor undergoing assisted reproduction and embryo transfer probably could benefit in assessing oocyte quality by measuring the levels of survivin expression in their granulosa cells. Therefore, if the survivin levels in granulosa cells are low, then ICSI should be concerned, as ICSI is an invasive method and good oocyte quality is not required. On the other hand, if survivin levels are highly expressed in granulosa cells then IVF should be preferred, as IVF is a non-invasive method and therefore normal sperm-egg interaction and good oocyte quality is essential [69].

## Conclusions

Expression of Oct-4 gene in luteinized granulosa cells seems to be at a ratio of 48%, and also seems to absent DAZL gene expression, which possibly suggests the existence of stem cells not originated from primordial germ cells. Absence of Oct-4 gene expression means probably the end of the productive journey of these cells, towards oocytes. Considering that Oct-4 transcripts could only be detected in 48%, any clinical significance seems to be limited. However, we found a clear clinical significance between the number of oocytes retrieved and expression levels of Oct-4 mRNA in granulosa cells. Therefore, when Oct-4 is expressed in granulosa cells appears to play an important role in the regulation of follicular growth during ART.

## Abbreviations

ART: Assisted reproduction technology; Oct-4: Octamer – binding transcription factor 4; DAZL: DAZ-like; IVF: In vitro fertilization; ICSI: Intracytoplasmic sperm injection; ET: Embryo transfer; FSH: Follicle-stimulating hormone; LH: Luteinizing hormone; GnRH: Gonadotropin releasing hormone; ART: Assisted reproductive technology; RRM: RNA recognition motif; RIA: Radio immuno assay; PRL: Prolactine; OHSS: Ovarian hyperstimulation syndrome; BMI: Body mass index; CMOCs: Cumulus–mature oocyte complexes; LIF: Leukemia-inhibiting factor.

## Competing interests

The authors declare that they have no competing interests.

## Authors’ contributions

MV was responsible for the original conception and design, edition of manuscript, supervision of whole attempt, data analysis and interpretation of the results. MV was responsible as well for correction, revision, and approval of the final version. TG performed all QC RT-PCR. MV, VK, CA and NP were involved in the drafting of the manuscript. All authors read and approved the final manuscript.
